# Age at Menarche, Schooling, and Sexual Debut in Northern Malawi

**DOI:** 10.1371/journal.pone.0015334

**Published:** 2010-12-09

**Authors:** Judith R. Glynn, Ndoliwe Kayuni, Sian Floyd, Emmanuel Banda, Monica Francis-Chizororo, Clare Tanton, Anna Molesworth, Joanne Hemmings, Amelia C. Crampin, Neil French

**Affiliations:** 1 Faculty of Epidemiology and Population Health, London School of Hygiene and Tropical Medicine, London, United Kingdom; 2 Karonga Prevention Study, Chilumba, Malawi; 3 Centre for Sexual Health and HIV Research, Research Department of Infection and Population Health, University College London, London, United Kingdom; University of Akron, United States of America

## Abstract

**Background:**

Age at sexual debut is a key behavioural indicator used in HIV behavioural surveillance. Early age at menarche may precipitate early sex through perceived readiness for sex, or through school drop-out, but this is rarely studied. We investigated trends and circumstances of sexual debut in relation to schooling and age at menarche.

**Methods and Findings:**

A cross-sectional sexual behaviour survey was conducted on all individuals age 15–59 within a demographic surveillance site in Karonga District, Malawi. Time trends were assessed using birth cohorts. Survival analysis was used to estimate the median age at menarche, sexual debut and first marriage. The 25^th^ centile was used to define “early” sex, and analyses of risk factors for early sex were restricted to those who had reached that age, and were done using logistic regression. Of the 8232 women and 7338 men resident in the area, 88% and 78%, respectively, were seen, and, 94% and 92% of these were interviewed. The median reported age at first sex was 17.5 for women and 18.8 for men. For women, ages at menarche, sexual debut and first marriage did not differ by birth cohort. For men, age at sexual debut and first marriage decreased slightly in later birth cohorts. For both men and women increased schooling was associated with later sexual debut and a longer delay between sexual debut and first marriage, but the associations were stronger for women. Earlier age at menarche was strongly associated with earlier sexual debut and marriage and lower schooling levels. In women early sexual debut (<16 years) was less likely in those with menarche at age 14–15 (odds ratio (OR) 0.31, 95%CI 0.26–0.36), and ≥16 (OR 0.04, 95%CI 0.02–0.05) compared to those with menarche at <14. The proportion of women who completed primary school was 46% in those with menarche at <14, 60% in those with menarche at 14–15 and 70% in those with menarche at ≥16. The association between age at menarche and schooling was partly explained by age at sexual debut. The association between age at menarche and early sex was not altered by adjusting for schooling.

**Conclusions:**

Women with early menarche start sex and marry early, leading to school drop-out. It is important to find ways to support those who reach menarche early to access the same opportunities as other young women.

## Introduction

Age at first sexual intercourse is one of the key sexual behaviour indicators recommended in second generation HIV surveillance [1]. Age at sexual debut has been shown to correlate with subsequent risk behaviour: on average those with younger ages at debut have more partners [2] and a higher risk of HIV [3]. Another important indicator of risk is the delay between sexual debut and first marriage, as this can be a time of high partner change, and is also associated with a higher number of partners later [4].

Largely missing from the discussion on risk factors for early sexual intercourse, at least in the biomedical literature, is age at menarche. Yet an association between early menarche and early sex has been noted where it has been measured [5], [6], [7]. Earlier menarche may lead to earlier sex because of the girl's desires but also due to social pressures and expectations. Girls who reach menarche may be regarded as “ready” to start sex and marry [8]. And for those who do start sex, pregnancy is more likely if they are physically mature.

There is particular interest in the role of schooling in sexual behaviour change [9], [10]. The association between level of schooling achieved and sexual debut is complex. Schooling is a measure of socio-economic status and of education itself. Socio-economic conditions which may limit school access or contribute to poor performance may also pre-dispose to early sexual activity [11]. And poor achievement may lead to drop out and sexual activity [11], [12]. For young women there is an additional complexity, since pregnancy may lead to school exclusion [11], [12]. Menarche may also precipitate early sexual debut through its affects on schooling. Menstruation can make school attendance difficult where sanitary arrangements are limited, leading to poor grades and school drop-out [13], [14].

Using data from a cross-sectional survey in a demographic surveillance site in northern Malawi we assessed secular trends in sexual debut and characteristics of first partners, and the links between menarche, schooling and sexual debut.

## Methods

The study was conducted as part of the Karonga Prevention Study in Karonga District, Malawi. A demographic surveillance system was set up in 2002 in a rural population of about 32,000 [15]. A biennial census started in 2004, which was replaced by annual census rounds, together with a socio-economic status update and HIV serosurveillance in 2007. A sexual behaviour survey was started in 2008, including all individuals aged 15–59. HIV prevalence in the area rose from less than 2% in the late 1980s to around 10% now [16].

Ethics statement: Ethics approval for the study was received from the Health Sciences Research Committee, Malawi, and the ethics committee of the London School of Hygiene & Tropical Medicine, UK. Before the start of the study the Traditional Authority that covers the area, and all village headmen and traditional advisors in the study area were informed about the aims of the study and the nature of the data to be collected, and their approval and verbal consent was sought. All household members were given a similar explanation and interviews were only conducted if verbal consent was given by the household head and by the respective household members. The consent for the demographic surveillance was recorded by the interview sheet being filled. Refusals were recorded in field registers. During the baseline census 15 households did not provide verbal consent and have consequently been excluded from the study. The socio-demographic data for this study come from the basic demographic surveillance for which the ethics committees agreed that written consent was not needed. For the sexual behaviour survey individual written consent was sought.

Schooling level achieved was asked for all individuals. In those aged up to 30 years at the time of interview, questions about schooling included the reason for leaving (asked as an open question: more than one reason could be recorded). Socio-economic status was only available at the time of interview, not historically. Parental education level, available for those aged up to 30 years at the time of interview, was used as a proxy of socio-economic status in adolescence.

Whether sexual debut occurred before menarche was asked throughout the study. A question on age at menarche was added in mid-October 2008, so was only available on about half of the women. Other questions included age at first intercourse, and information on characteristics of the first sex partner, and age at first marriage.

### Statistical analysis

Data from the first round of the sexual behaviour survey were used. Median age at menarche, first sexual intercourse and first marriage were determined using survival analysis, to allow for right censoring of those who had not yet experienced these events. The data were smoothed by adding a random fraction of a year, since age in whole years was recorded for events, not dates [17], [18]. The 25^th^ centile of age at sexual debut was used to define “early” sex, and subsequent analyses of determinants of early sex only included individuals over these cut-off ages, and was done using logistic regression. A delay between sexual debut and marriage was defined as “long” if the age at first marriage (or the current age for those not yet married) was more than one year older than the age at first sex, and analysis was restricted to individuals more than one year older than their age at first sex. This period of delay was chosen to distinguish marriage happening soon after, and perhaps related to, sexual debut from that happening later.

The analysis explored secular trends (using birth cohorts, <1965, 1965–74, 1975–84, 1985–94) in ages at first sexual intercourse and first marriage and in partner types; and risk factors for early sex and for a long delay between first sex and first marriage. For women, analyses also explored the relationship between age at menarche, schooling and sexual debut. In particular we assessed whether an association between age at menarche and sexual debut was affected by adjusting for schooling (which would suggest that schooling was on the causal pathway between menarche and sexual debut); and whether an association between age at menarche and schooling was affected by adjusting for sexual debut and marriage (which would suggest that an association between menarche and schooling was mediated via sexual debut and/or marriage).

## Results

At the time of the survey there were 8232 women and 7338 men aged 15–59 resident within the demographic surveillance area. 987 (12%) women and 1613 (22%) men were not found and seen by the interviewers. Of those who were seen 6825 (94%) women and 5283 (92%) men agreed to be interviewed about their sexual behaviour, and 6796 women and 5253 men were interviewed.

**Figure 1:**
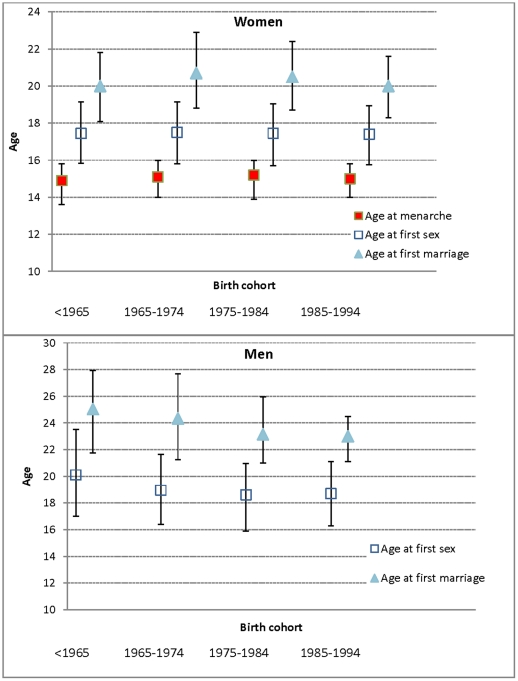
Median age at first sexual intercourse and first marriage for women and men. Graphs show median and interquartile range.

At the time of interview 89% of the women had ever had sexual intercourse, and 85% had ever been married. Equivalent figures for the men were 80% and 62%. Median age at first sexual intercourse for the women was 17.5, and for first marriage was 18.5 years. Using birth cohorts, both values were unchanged over time (figure 1). For the men the median age at first sexual intercourse was 18.8 years, and at marriage 23.7 years: both sexual debut and marriage occurred at slightly younger ages in the more recent birth cohorts (figure 1).

Using the 25^th^ centile as the cut-off, early sex was defined as sexual debut at <16 years for women and <17 years for men.

### Women

There was a strong association between age at menarche and age at sexual debut, with 55% of those with menarche at <14 years having had early sex, compared with 27% of those with menarche at 14 or 15, and 4% of those with menarche aged ≥16 years (table 1). Sexual debut before menarche was unusual: it was reported by 2.8% of women overall, but the proportion increased over time, from 1.3% in those born before 1965 to 4.1% in those born 1985–94 (*p* <0.001) (table 2). The median interval between menarche and sexual debut was 3.5 years for those with menarche age <14, 2.7 years for those with menarche at 14–15, and 2.5 years for those with menarche at 16 or older. The association between age at menarche and early sex was not changed by adjusting for schooling or birth cohort (table 1).

**Table 1:** Risk factors for early sexual debut (sex at <16 years for women and <17 years for men).

				Women				Men	
		n/N	%	OR (95% CI)	aOR (95% CI)	n/N	%	OR (95% CI)	aOR (95% CI)
All		1874/6334	29.6			1399/4556	30.7		
Birth cohort									
	<1965	277/1000	27.7	1	1^1^	179/714	25.1	1	1^1^
	1965–74	361/1253	28.8	1.1 (0.88–1.3)	1.3 (1.1–1.6)	258/863	29.9	1.3 (1.0–1.6)	1.3 (1.1–1.7)
	1975–84	640/2066	31.0	1.2 (0.99–1.4)	1.7 (1.4–2.0)	501/1529	32.8	1.5 (1.2–1.8)	1.6 (1.3–1.9)
	1985–94	596/2015	29.6	1.1 (0.93–1.3)	1.7 (1.4–2.0)	461/1450	31.8	1.4 (1.1–1.7)	1.5 (1.2–1.8)
Schooling									
	None/P1–5	439/1022	43.0	1	1^2^	149/451	33.0	1	1^2^
	P6–7	648/1609	40.3	0.90 (0.76–1.0)	0.81 (0.68–0.95)	248/684	36.3	1.2 (0.90–1.5)	1.1 (0.86–1.4)
	P8	494/1902	26.0	0.47 (0.40–0.55)	0.42 (0.35–0.49)	441/1323	33.3	1.0 (0.81–1.3)	1.0 (0.81–1.3)
	Secondary+	231/1595	14.5	0.22 (0.19–0.27)	0.19 (0.15–0.23)	532/1987	26.8	0.74 (0.59–0.92)	0.7 (0.56–0.87)
Age at menarche									
	<14	450/813	55.4	1	1^3^				
	14–15	458/1669	27.4	0.31 (0.26–0.36)	0.32 (0.26–0.38)				
	≥16	29/693	4.2	0.04 (0.02–0.05)	0.04 (0.03–0.06)				
Among age ≤30									
Mother schooling									
	≤Primary	893/2942	30.4	1	1^4^	704/2203	32.0	1	1^4^
	Secondary	106/397	26.7	0.83 (0.66–1.1)	0.84 (0.66–1.1)	80/237	33.8	1.1 (0.92–1.4)	1.1 (0.82–1.4)
Father schooling									
	≤Primary	689/2100	32.8	1	1^4^	535/1648	32.5	1	1^4^
	Secondary	291/1162	25.0	0.69 (0.59–0.81)	0.69 (0.59–0.81)	226/735	30.8	0.92 (0.77–1.1)	0.93 (0.77–1.1)

OR = odds ratio, aOR = adjusted odds ratio.

1 Adjusted for schooling.

2 Adjusted for birth cohort.

3 Adjusted for birth cohort and schooling.

4 Adjusted for current age.

**Table 2:** Characteristics of sexual debut by birth cohort.

		Birth cohort				
		<1965	1965–74	1975–84	1985–94	*P*
**Women**						
Before menstruation	N	997	1202	2001	1551	
	% (n)	1.3 (13)	2.6 (31)	2.6 (52)	4.1 (64)	<0.001
Type of partner	N	1035	1276	2089	1624	
Husband	% (n)	73.3 (759)	59.2 (755)	48.4 (1010)	42.1 (683)	
Boyfriend	% (n)	24.5 (254)	39.2 (500)	49.6 (1036)	55.9 (907)	
Other/unknown	% (n)	2.1 (22)	1.7 (21)	2.1 (43)	2.1 (34)	<0.001
Married 1^st^ partner (if ever married)	N	1026	1253	2046	1422	
	% (n)	84.2 (864)	76.3 (956)	70.3 (1439)	71.2 (1012)	<0.001
Condom 1^st^ sex if not husband	N	271	513	1063	933	
	% (n)	0 (0)	2.0 (10)	16.8 (179)	41.2 (384)	<0.001
**Men**						
Type of partner	N	733	886	1513	1085	
Wife	% (n)	29.1 (213)	21.4 (190)	15.5 (235)	6.0 (65)	
Girlfriend	% (n)	67.1 (492)	74.6 (661)	79.6 (1205)	86.4 (937)	
Other/unknown	% (n)	3.8 (28)	4.0 (35)	4.8 (73)	7.7 (83)	<0.001
						
Married 1^st^ partner (if ever married)	N	721	866	1324	332	
	% (n)	37.6 (271)	30.4 (263)	29.9 (396)	32.8 (109)	0.003
Condom 1^st^ sex if not wife	N	514	690	1262	1009	
	% (n)	0.7 (5)	5.0 (36)	40.8 (292)	53.5 (383)	<0.001

Age at menarche was similar in each birth cohort (median 15.1 years, figure 1). The interval between age at menarche and age at sexual debut was also similar in each birth cohort: median 2.8, 3.0, 2.9, 2.8 years for the 4 birth cohorts. There was weak evidence of effect modification between the associations of age at menarche and birth cohort with age at sexual debut (*p* lrtest for interaction 0.2). In those with menarche at 14 years or older there was no association between birth cohort and early sex, but in those with early menarche, the likelihood of early sex increased with later birth cohort (48.1%, 47.3%, 57.1% 60.4%, for the 4 birth cohorts respectively, *p* = 0.03).

Early sex was more common in those with less schooling. It was also more common in those whose parents had had less schooling (only asked for those aged 30 and under). Although overall there was no association between birth cohort and early sex, after adjusting for schooling level, early sex was more likely in those born more recently. There was no evidence of effect modification: within each level of schooling, there was a trend towards earlier sex in the younger cohorts. In those aged ≤30 years, the association of schooling and early sex was not altered by adjusting for parental schooling level. In this age group, 45% gave pregnancy or marriage as a reason for leaving school, and 33% were still in school. Those at higher levels of schooling were more likely to give pregnancy or marriage as the reason for leaving than those with more basic schooling. Menstruation was never mentioned as a reason for leaving school.

Among the oldest women 73% said their first sex partner was their husband, compared to 42% of the youngest women (table 2) The proportion reporting that their first partner was a boyfriend increased over time, from 25% to 56%. Many women later married this boyfriend but among ever married women there was still a decrease by birth cohort in the proportion who married their first sex partner (table 2). Condom use with the first partner, excluding those whose first partner was their husband, rose from 0% in the oldest women to 41% in the youngest cohort.

A quarter of the women delayed more than one year between sexual debut and marriage. Delaying more than a year was less common in those born before 1965, but was similar in the other birth cohorts. Delay was more common in those with more schooling and in those with early sexual debut, and all these associations persisted after adjusting for each other (table 3). The estimates were similar when also adjusted for age at menarche, and, in those 30 and under, when adjusted for parental education. Delay was longer in those with later menarche, and this association was stronger after adjustment for age at sexual debut and weaker after adjustment for schooling.

**Table 3:** Factors associated with delay of more than one year between sexual debut and marriage, restricted to those seen more than one year since sexual debut.

				Women				Men	
		n/N	%	OR	aOR	n/N	%	OR	aOR
All		1521/5479	27.8			2809/3749	74.9		
Birth cohort									
	<1965	150/988	15.2	1	1^1^	438/706	62.0	1	1^1^
	1965–74	321/1244	25.8	1.9 (1.6–2.3)	1.7 (1.3–2.1)	598/844	70.9	1.5 (1.2–1.8)	1.3 (0.99–1.6)
	1975–84	627/2034	30.8	2.5 (2.0–3.0)	1.7 (1.4–2.1)	1061/1427	74.4	1.8 (1.5–2.2)	1.4 (1.1–1.7)
	1985–94	423/1213	34.9	3.0 (2.4–3.7)	1.8 (1.4–2.2)	712/772	92.2	7.3 (5.4–9.8)	3.7 (2.7–5.2)
Schooling									
	None/P1–5	170/970	17.5	1	1^1^	250/367	68.1	1	1^1^
	P6–7	327/1421	23.0	1.4 (1.1–1.7)	1.3 (1.1–1.7)	373/542	68.8	1.0 (0.78–1.4)	0.85 (0.61–1.2)
	P8	439/1675	26.2	1.7 (1.4–2.0)	1.9 (1.5–2.3)	844/1165	72.5	1.2 (0.95–1.6)	1.5 (1.1–2.0)
	Secondary+	534/1237	43.2	3.6 (2.9–4.4)	4.5 (3.6–5.7)	1271/1584	80.2	1.9 (1.5–2.4)	2.5 (1.9–3.4)
Early sex									
	No	814/3636	22.4	1	1^1^	1444/2323	62.2	1	1^1^
	Yes	699/1832	38.2	2.1 (1.9–2.4)	2.9 (2.5–3.3)	1309/1370	95.6	13.1 (10.0–17.1)	12.6 (9.5–16.6)
Age at menarche									
	<14	215/758	28.4	1	1^1^				
	14–15	446/1399	31.9	1.2 (0.97–1.4)	1.5 (1.2–1.9)				
	≥16	192/575	33.4	1.3 (1.0–1.6)	2.1 (1.6–2.8)				
Mother schooling									
	≤Primary	745/2274	32.8	1	1^2^	1260/1533	82.2	1	1^2^
	Secondary	117/290	40.3	1.4 (1.1–1.8)	1.5 (1.1–1.9)	143/164	87.2	1.5 (0.92–2.4)	1.4 (0.85–2.4)
Father schooling									
	≤Primary	489/1634	29.9	1	1^2^	922/1153	80.0	1	1^2^
	Secondary	346/870	39.8	1.5 (1.3–1.8)	1.7 (1.4–2.0)	443/494	89.7	2.2 (1.6–3.0)	2.3 (1.7–3.3)

OR = odds ratio, aOR = adjusted odds ratio.

1 Adjusted for early sex, birth cohort and schooling level.

2 Adjusted for age and early sex.

There was a strong association between age at menarche and school level achieved (table 4). Standard 8, the end of primary school, was reached by 46% (383/836) of women with menarche <14 years, 60% (1024/1713) of those with menarche at 14 or 15, and 70% (476/680) with menarche at ≥16 years, giving odds ratios, compared to those with menarche at <14, of 1.7 (1.5–2.0) and 2.7 (2.2–3.4) for those with menarche at 14/15 and ≥16 years respectively. This association was unchanged when adjusted for birth cohort, but was partly explained by adjusting for age at sexual debut (aOR 1.5 (1.2–1.8) for age at menarche of 14/15 and aOR 1.7 (1.3–2.1)for age at menarche ≥16, compared with those with menarche at <14 years old), and further when also adjusted for age at first marriage (aOR 1.3 (1.1–1.6) and 1.3 (1.0–1.8) for age at menarche of 14/15 and ≥16 respectively, table 4).

**Table 4:** Associations with age at menarche.

		Age at menarche	
	<14	14–15	≥16
Schooling level			
N	836	1713	680
None/P1–5 (%)	18.2	12.8	11.3
P6–7 (%)	35.7	27.4	18.7
P8 (%)	27.6	34.5	30.9
Secondary+ (%)	18.5	25.3	39.1
Age at sexual debut			
N	863	1761	694
median, IQR	15.7 (14.6–17.8)	17.3 (15.9–18.8)	18.9 (17.9–20.6)
Age at first marriage			
N	866	1774	704
median, IQR	16.9 (15.5–18.9)	18.5 (16.9–20.1)	20.3 (18.8–21.9)
Reason for leaving school (if age ≤30)	540	1124	406
Pregnancy/marriage	57.2	49.2	49.5
other	25.0	24.1	28.3
In school	17.8	27.7	22.2
Finished primary school	46% (383/836)	60% (1024/1713)	70% (476/680)
OR for finishing primary	ref	1.7 (1.5–2.0)	2.7 (2.2–3.4)
OR among sexually active	ref	1.8 (1.5–2.1)	2.7 (2.1–3.3)
OR adjusted for age at first sex	ref	1.5 (1.2–1.8)	1.7 (1.3–2.1)
OR adjusted for age at first sex among ever married	ref	1.4 (1.2–1.7)	1.6 (1.2–2.1)
OR adjusted for age at first sex and marriage	ref	1.3 (1.1–1.6)	1.4 (1.0–1.8)

OR = odds ratio.

### Men

Early sex (before age 17) was less common in the oldest cohort and in those with secondary or more schooling (table 1). These associations did not change when adjusted for each other. Among men aged 30 and under, 8% reported that they had left school because of marriage or because their girlfriend or wife was pregnant. The proportion of men who reported that their first partner was their wife decreased from 29% in the oldest group to 6% in the youngest (table 2). Most of the other first partners were described as girlfriends, and there was only a slight decrease over time in the proportion who married their first sex partner (from 38% to 33%). Condom use with the first partner (excluding spouses) rose to 54% in the youngest cohort.

Most men (75%) delayed more than a year between sexual debut and first marriage (table 3). Delay was more common in the more recent cohorts and among those with more schooling, and much more common (96%) in those with early sexual debut.

Among men aged 30 and under, there was no association between parental education and early sex (table 1), but those whose fathers had had secondary schooling were more likely to delay between sexual debut and first marriage (table 3). Adjusting for parental education made little difference to the associations between a man's own schooling and either early sex or the delay between sexual debut and first marriage.

## Discussion

For women there was little change over calendar time in age at menarche, sexual debut or first marriage. For men, there was a slight decrease in age at debut and first marriage, and an increase in delay between sexual debut and marriage. Changes over time were more marked in the type of first partner, with a decrease in the proportion reporting that sexual debut occurred within marriage for both men and women. Condom use with the first partner, where this was not the spouse, rose to 54% and 41% for the youngest groups of men and women. Using these cross-sectional data, secular trends can only be examined using birth cohorts. These will under-represent the higher risk individuals in older age groups, some of whom will have died of HIV. This may account for the downward shift in age at sexual debut for men, and obscure any trends for women.

The results relied on recall of age at events. Several studies have examined the accuracy of recall of age at sexual debut and first marriage by comparing results from the same individual over survey rounds. These have shown that 30–50% of reports are inconsistent, but there are no particular trends towards under or over estimation of ages, so aggregate results are unbiased [4], [18], [19]. In the individual level analyses, however, the misclassification will tend to underestimate any associations seen, so the true strength of the associations with schooling and menarche may be stronger.

For both men and women, those who achieved a higher level of schooling had later sexual debut and a longer delay between sexual debut and first marriage. The associations were much stronger for women. Schooling is also a marker of socio-economic status. We did not know socio-economic status at the time of sexual debut or marriage, but the associations found with an individual's own schooling were not altered by adjusting for parental education status, which is a proxy for socio-economic status early in life. While the level of parental schooling may not be very accurately known or recalled, and thus there could be residual confounding, this suggests that the associations are with schooling itself.

The extent to which education influences behaviour or behaviour influences education is unclear, but it is likely that the effect is in both directions. Nearly half the women (and 8% of the men) aged 30 and under gave pregnancy or marriage as the reason for leaving school. This is a high percentage compared with other reports from sub-Saharan Africa [11]. From antenatal clinic (ANC) surveys in the same setting, 31% of teenage women attending ANC with their first pregnancy were at school when they became pregnant. 8% of teenage women attending ANC with subsequent pregnancies had also been at school when they became pregnant, confirming that some women are able return to education after the birth of a child (unpublished data).

For women, the onset of menstruation may be a major – and neglected – factor influencing both schooling and sexual debut. Menstruation marks physical maturity, the transition to womanhood, and, in some cultures, is marked by initiation rituals. In many and diverse settings girls are seen as “ready” for sexual activity soon after menarche (and boys as “needing” sexual activity once pubescent) [20]. Menstruation also brings practical problems with school attendance in managing the blood loss where latrine facilities are poor, disposable pads are unaffordable, and it is difficult to wash in private [14]. Girls may skip school rather than risk discovery [13], [14]. Physical maturity can also bring unwanted attention and teasing from boys, adding to the problems of school attendance [8], [13]. An association between having reached puberty and school drop-out has been found previously [21].

Early menarche could therefore lead to early sexual debut via school drop-out, or more directly, in response to individual and societal pressures. While there is no scope for intervention in the timing of menarche, there are possibilities for intervening on the consequences. It is therefore important to understand the dominant pathways. If the practical problems of menstruation and schooling are the key, then the emphasis should be on improving the facilities and attitude of schools [5], [8], [13]. There are already some initiatives to do that internationally (eg UNICEF [22]) and nationally (Lieza du Preez, personal communication). However, if the direct pathway, through individual and societal pressures, is prominent, reducing early sexual debut following menarche will also require a shift in expectations.

The median age at menarche of 15 years is the same as measured previously in northern Malawi [23]. In Karonga District, unlike southern Malawi [23], [24], there are no initiation rituals. At menarche girls are traditionally sent to stay with an aunt or other female relative, for instruction, and it is likely to become known in the community. In the current analysis there was a strong association between age at menarche and school level reached, consistent with an effect of menstruation on schooling. But this association was much less strong after adjusting for early sexual debut, suggesting that it was the early sex that led to the lower schooling level, not primarily the menstruation itself. Conversely the association between age at menarche and early sexual debut was not changed by adjusting for schooling level. This suggests that the major pathway by which earlier menarche leads to earlier sexual debut is not through the effect on schooling.

The perception that girls are “ready” for sex and marriage at puberty prevails in Karonga District, at least traditionally (unpublished results). And the interval between menarche and sexual debut was similar in the different birth cohorts, so this perception may not have changed. Whatever the causes, the effects are very large. More than half the girls with menarche before 14 fail to finish primary school, have sex before they are 16 and are married before 17, whereas 70% of girls with menarche at age 16 or older finish primary school, many going on to secondary education, start sex after the age of 18 and marry after the age of 19. The schooling level reached by girls with late menarche is similar to that of boys in the community. That reached by girls with earlier menarche falls far short of this.

Age at puberty is falling in many societies [25]. It will become increasingly important to find ways to stop girls who reach menarche early from being disadvantaged for the rest of their lives.
